# Inflammasome-independent IL-1β activation *via* staphopain A protease of *Staphylococcus aureus*

**DOI:** 10.1016/j.jbc.2025.110574

**Published:** 2025-08-08

**Authors:** Stefan Bauernfried, Tobias Komar, Katja Sterle, Maria C. Tanzer, Alexander R. Horswill, Matthias Mann, Veit Hornung

**Affiliations:** 1 Gene Center and Department of Biochemistry, Ludwig-Maximilians-Universität, Munich, Germany; 2 Department of Proteomics and Signal Transduction, Max Planck Institute of Biochemistry, Martinsried, Germany; 3 Walter and Eliza Hall Institute of Medical Research, Parkville, Victoria, Australia; 4 Department of Medical Biology, University of Melbourne, Parkville, Victoria, Australia; 5 Department of Immunology and Microbiology, University of Colorado Anschutz Medical Campus, Aurora, Colorado, USA; 6 Novo Nordisk Foundation Center for Protein Research, Faculty of Health and Medical Sciences, University of Copenhagen, Copenhagen, Denmark

**Keywords:** interleukin-1β, NLRP1, inflammasome, *Staphylococcus aureus*, keratinocytes, staphopain A, microbial proteases

## Abstract

Interleukin-1β (IL-1β) is a pivotal mediator of innate immunity, essential for orchestrating the acute inflammatory response. While the canonical activation of IL-1β involves cleavage of its inactive precursor (pro-IL-1β) by the inflammatory cysteine protease caspase-1, certain bacterial proteases, such as those secreted by group A *Streptococcus* and *Pseudomonas aeruginosa*, can also activate pro-IL-1β. In this study, we demonstrate that infection of human N/TERT-1 immortalized keratinocytes by *Staphylococcus aureus* induces IL-1β processing independently of the classical inflammasome pathways. Biochemical analysis reveals that a secreted factor from *S. aureus* cleaves pro-IL-1β at a site proximal to the canonical caspase-1 cleavage site, rendering the cytokine bioactive. Specifically, we identify the secreted cysteine protease staphopain A as responsible for this cleavage. Our findings highlight a novel mechanism of inflammasome-independent IL-1β activation through microbial proteases, expanding the understanding of pathogen–host interactions in immune responses, specifically in the skin.

Interleukin-1β (IL-1β) is a key mediator of innate immunity and inflammation and plays a critical role in orchestrating acute inflammation (1, 2). As such, IL-1β signaling triggers induction of cytokines and chemokines, leukocyte infiltration, T-cell activation, and acute phase protein synthesis (3, 4). At the same time, chronic IL-1β activation is implicated in autoimmune diseases, such as inflammatory bowel disease, rheumatoid arthritis, and systemic lupus erythematosus, as well as rare, hereditary autoinflammatory disorders, including cryopyrin-associated periodic syndromes and familial Mediterranean fever (5).

IL-1β is expressed as an inactive precursor (pro-IL-1β) that requires proteolytic cleavage to be rendered biologically active. Canonically, the inflammatory cysteine protease caspase-1 processes pro-IL-1β, thereby releasing the mature cytokine (6, 7, 8). Caspase-1 is activated by proximity-induced dimerization of its protease domains upon recruitment to high-molecular-weight signaling complexes, known as inflammasomes (9). Among inflammasome-forming sensors, nucleotide-binding domain leucine-rich repeat (NLR) protein NLRP1 was the first to be characterized as such (10). Human NLRP1 is activated by inhibition of dipeptidyl peptidases 8 and 9, viral proteases through functional degradation of the N terminus, dsRNA, as well as UV B- and toxin-induced ribotoxic stress response (11, 12, 13, 14, 15, 16). Active caspase-1 not only processes pro-IL-1β into its bioactive form but also cleaves gasdermin D (GSDMD), which mediates the lytic cell death of pyroptosis (17, 18, 19).

Despite its canonical processing by caspase-1, pro-IL-1β can also be rendered bioactive by several additional host-encoded proteases independent of inflammasome activation. Indeed, neutrophil enzymes, such as proteinase-3 and elastase, cleave the precursor protein at a site proximal to the canonical cleavage site of caspase-1, thereby generating active IL-1β (20, 21, 22). Further, granzyme A, a serine protease expressed in granules of cytotoxic T lymphocytes and natural killer cells, has been described to convert pro-IL-1β into its mature form (23). In addition, caspase-8 activated downstream of Toll-like receptor 3 and 4 signaling has been shown to cleave IL-1β (24).

Interestingly, during the initial search for the host protease responsible for converting pro-IL-1β, it was also observed that a protease derived from *Staphylococcus aureus* could process pro-IL-1β into its active cytokine form *in vitro*. However, this processing produced an N-terminally extended variant of IL-1β, distinct from the primary mature form typically generated by host cells. In these early studies, the identity of this protease remained unclear, and further exploration in this direction was not pursued (25), potentially because of subsequent findings that *S. aureus* was a potent activator of the NLRP3 inflammasome in myeloid cells (see later). Similarly, the group A *Streptococcus*–encoded protease SpeB (streptococcal pyrogenic exotoxin B) was shown to cleave pro-IL-1β and generate mature IL-1β in a caspase-1-independent manner, both *in vitro* (26) and *in vivo* (27). Peptide sequencing revealed that the cleavage site of group A *Streptococcus* SpeB is located after phenylalanine residue 105, which is 11 amino acids N-terminal to the canonical caspase-1 cleavage site (27). In addition, *Pseudomonas aeruginosa* was reported to mature IL-1β independently of inflammasome activation during infection of both murine and human cells, a process that relies on its encoded metalloprotease LasB (28). As noted previously, after the delineation of the inflammasome pathway, *S. aureus* was found to robustly activate the NLRP3 inflammasome in both human and mouse monocytic cells through its pore-forming hemolysins (29, 30). This pore formation disrupts the plasma membrane, leading to potassium efflux, a well-established upstream trigger for NLRP3 activation (31).

In humans, *S. aureus* is a major pathogen and a leading cause of skin and soft tissue infections (32). However, whether *S. aureus* infection directly induces inflammasome activation and subsequent IL-1β maturation in human keratinocytes remains unclear.

## Results

### *S. aureus* infection leads to processing of pro-IL-1β independent of the classic inflammasome cascade

**Figure 1:**
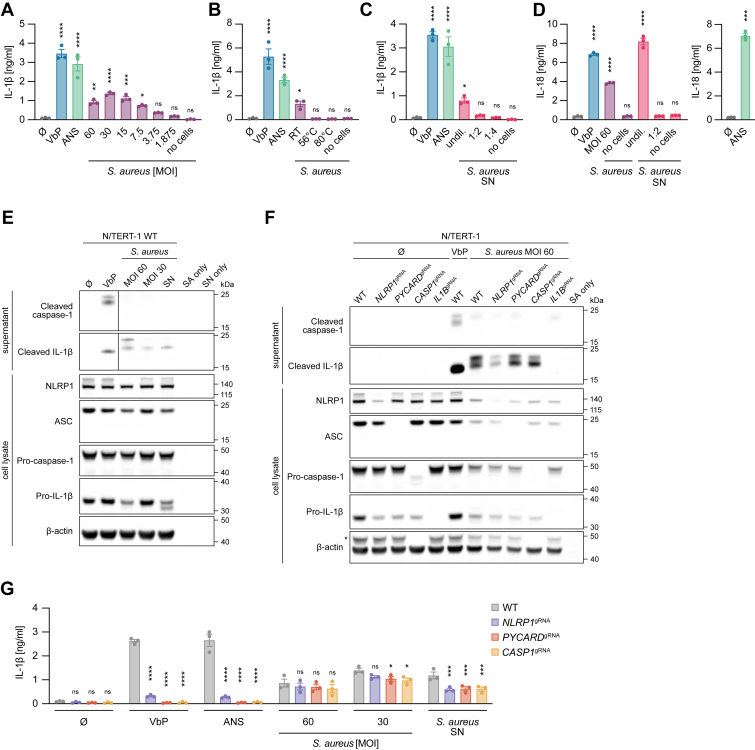
***Staphylococcus aureus* infection leads to release and processing of pro-IL-1β independent of the classic inflammasome cascade.***A*–*C*, unmodified keratinocytes were left untreated, stimulated with VbP or ANS for 14 h or infected with *S. aureus* at the indicated MOIs (*A*), infected at MOI 60 after being incubated at the indicated temperature for 30 min (*B*), or stimulated with supernatant (SN) of an overnight sterile-filtered *S. aureus* ATCC 6538 culture (*S. aureus* SN) (*C*). Keratinocytes were infected with *S. aureus* for 2 h followed by 16 h of gentamicin treatment. *S. aureus* SN stimulation was performed for 18 h. Bacteria at MOI 60 or undiluted *S. aureus* SN without keratinocytes (no cells) were used as a control. IL-1β release was determined. *D*, unmodified keratinocytes were left untreated, stimulated with VbP for 15 h or ANS for 3 h, infected with *S. aureus* at MOI 60 for 14 h followed by 1 h of gentamicin treatment, or stimulated with *S. aureus* SN for 15 h. Bacteria at MOI 60 or undiluted *S. aureus* SN without keratinocytes (no cells) were used as a control. IL-18 release was determined. *E*, unmodified keratinocytes were left untreated, stimulated with VbP for 24 h, infected with *S. aureus* at the indicated MOIs for 7 h followed by 1 h of gentamicin treatment, or stimulated with *S. aureus* SN for 8 h. Bacteria (SA only) or *S. aureus* SN (SN only) without keratinocytes were used as a control. Lysates or SNs were blotted for NLRP1, ASC, caspase-1, IL-1β, or β-actin. SNs of untreated and VbP-treated keratinocytes were imaged at a shorter exposure than other samples in these rows (separated by a *vertical line*) because of different signal intensities. *F*, keratinocytes with the indicated genotypes were left untreated, stimulated with VbP for 24 h, or infected with *S. aureus* at MOI 60. *S. aureus* infection was performed as in (*E*). Bacteria (SA only) without keratinocytes were used as a control. Lysates or SNs were blotted for NLRP1, ASC, caspase-1, IL-1β, or β-actin. *Asterisk* denotes bands from previous blotting for caspase-1. *G*, keratinocytes with the indicated genotypes were left untreated, stimulated with VbP or ANS, infected with *S. aureus* at the indicated MOIs, or stimulated with *S. aureus* SN. *S. aureus* infection and SN stimulation were performed as in (*A*–*C*). IL-1β release was determined. Data are depicted as mean ± SEM of *n* = 3 independent experiments (*A*–*D*, and *G*) or as one representative experiment out of two (*E* and *F*). Statistical analysis was conducted by one-way ANOVA with Dunnett’s multiple comparison correction (*A*, *B*, *C*, and *D* [*left panel*]), two-way ANOVA with Dunnett’s multiple comparison correction (*G*), or unpaired, two-tailed Student’s *t* test (*D*, *right panel*). Comparisons in (*A*–*D*) were made against the untreated control, whereas comparisons in (*G*) were made against the WT control. ∗∗∗∗*p* < 0.0001, ∗∗∗*p* < 0.001, ∗∗*p* < 0.01, and ∗*p* < 0.05; ns, not significant. ANS, anisomycin; IL-18, interleukin-18; IL-1β, interleukin-1β; MOI, multiplicity of infection; pro-IL-1β, inactive precursor of IL-1β; VbP, Val-boroPro.

Recent studies have shown that, in addition to classic immune cells, such as macrophages, nonimmune cells like keratinocytes also express innate immune sensors, specifically inflammasomes, and contribute to the immune response (14, 33, 34). To study *S. aureus* infection in human keratinocytes, the predominant cell type in the epidermis, we used human N/TERT-1 immortalized keratinocytes as our model system. Treatment of human N/TERT-1 immortalized keratinocytes with the DPP8/9 inhibitor, Val-boroPro (VbP), or the ribotoxic stress response inducer, anisomycin (ANS), both activated the NLRP1 inflammasome and led to the secretion of IL-1β, as measured by IL-1β ELISA (Fig. 1*A*). Infection of human keratinocytes with the *S. aureus* subsp. *aureus* Rosenbach strain ATCC 6538 likewise resulted in the release of IL-1β across various multiplicities of infection (MOIs) (Fig. 1*A*). In contrast, exposure of keratinocytes to heat-inactivated *S. aureus* did not result in significant IL-1β release, suggesting that live *S. aureus* is required for inflammasome activation (Fig. 1B). Interestingly, the supernatant (SN) from *S. aureus* cultures was capable of inducing IL-1β release as well (Fig. 1C), indicating that soluble factors released by *S. aureus* into the culture medium can trigger inflammasome activation, even in the absence of live bacteria. Notably, dilution of the SN resulted in a reduction in activity greater than expected based on dilution alone, possibly because of the presence of an inhibitory factor in the culture medium that is normally metabolized by *S. aureus*. In addition to IL-1β release, infection of keratinocytes with *S. aureus* and stimulation with *S. aureus* SN also triggered secretion of IL-18 (Fig. 1*D*). However, stimulation of keratinocytes with *S. aureus* did not induce classic markers of inflammasome activation, as evidenced by the absence of cleaved caspase-1 in the SN of stimulated cells (Fig. 1*E*). Moreover, while cleaved IL-1β was detected, it exhibited a distinct cleavage pattern with two bands and a higher apparent molecular weight than caspase-1-cleaved IL-1β. To determine if pro-IL-1β cleavage could occur independently of inflammasome components, such as the inflammasome sensor NLRP1, the adaptor protein ASC, or caspase-1, we infected keratinocytes deficient in these components. Doing so, we still observed IL-1β release by immunoblot and ELISA (Fig. 1, *F* and *G*). This implies the presence of a secreted *S. aureus* factor that can either induce host-dependent cleavage of pro-IL-1β at a unique site different from the canonical caspase-1 cleavage site or a factor that directly cleaves pro-IL-1β.

### Pro-IL-1β is cleaved by a secreted *S. aureus* factor and is bioactive

**Figure 2:**
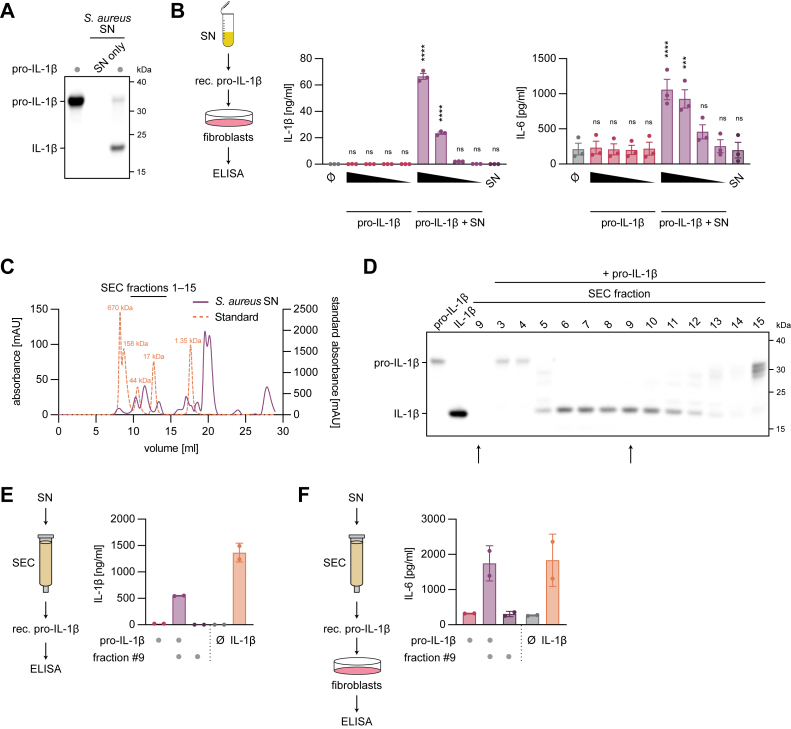
**Pro-IL-1β is cleaved by a secreted *Staphylococcus aureus* factor and is thus rendered bioactive.***A*, recombinant human pro-IL-1β (rec. pro-IL-1β) was left untreated or incubated with *S. aureus* SN for 8 h at 37 °C. *S. aureus* SN alone (SN only) was used as a control. Samples were blotted for IL-1β. *B*, MeWo fibroblasts were left untreated or stimulated with rec. pro-IL-1β, rec. pro-IL-1β pretreated with *S. aureus* SN, or SN alone. Decreasing concentrations (1:10, 1:30, 1:300, and 1:3000 dilution) of uncleaved and cleaved pro-IL-1β were used. IL-1β and IL-6 levels were determined after 15 h. *C*, concentrated *S. aureus* SN was subjected to size-exclusion chromatography (SEC) using a Superdex 75 10/300 GL column. Chromatogram of *S. aureus* SN and protein standards is depicted. SEC fractions 1 to 15 are highlighted. *D*, rec. pro-IL-1β was left untreated or incubated with indicated SEC fractions from (*C*) for 8 h at 37 °C. Rec. mature IL-1β and SEC fraction 9 alone were used as a control. Samples were blotted for IL-1β. Conditions with SEC fraction 9 alone and fraction 9 pretreated with rec. pro-IL-1β are highlighted. *E*, *S. aureus* SN was fractionated by SEC (*C*) and incubated with rec. pro-IL-1β for 8 h at 37 °C (*D*). IL-1β levels of rec. pro-IL-1β, rec. pro-IL-1β incubated with SEC fraction 9, SEC fraction 9 alone, or rec. mature IL-1β were determined. Of note, the data are extracted from Fig. S1*B* and are therefore identical to the data presented in Fig. S1*B*. *F*, MeWo fibroblasts were left untreated or stimulated with cleavage products from (*E*). IL-6 levels were determined after 8 h. Of note, the data are extracted from Fig. S1*C* and are therefore identical to the data presented in Fig. S1*C*. Data are depicted as mean ± SEM of *n* = 3 independent experiments (*B*), as one representative experiment out of two (*A*, *C*, and *D*), or as mean ± SD of *n* = 2 independent experiments (*E* and *F*). Statistical analysis was conducted by one-way ANOVA with Dunnett’s multiple comparison correction (*B*). Comparisons in (*B*) were made against the untreated control. ∗∗∗∗*p* < 0.0001, ∗∗∗*p* < 0.001; ns, not significant. Pro-IL-1β, inactive precursor of IL-1β; SN, supernatant.

One possible mechanism for IL-1β cleavage could involve a secreted *S. aureus* protease. To test this hypothesis, we incubated recombinant pro-IL-1β with *S. aureus* SN and assessed its processing by immunoblot. Incubation of pro-IL-1β with *S. aureus* SN showed a band for IL-1β running at less than 25 kDa, indicating a cleavage event (Fig. 2*A*). To assess whether this species is bioactive and capable of signaling through its cognate receptor, we treated MeWo fibroblasts with either untreated pro-IL-1β or pro-IL-1β pretreated with *S. aureus* SN and measured IL-6 secretion as an indicator of IL-1 signaling (Fig. 2*B*). These experiments demonstrated that only pro-IL-1β treated with *S. aureus* SN, but not untreated pro-IL-1β or *S. aureus* SN alone, was biologically active in terms of inducing IL-6 production (Fig. 2*B*, *right panel*). In line with pro-IL-1β being processed by *S. aureus* SN, the mature form IL-1β detected in these SNs correlated well with the IL-6 response (Fig. 2*B*, *left panel*). To identify the secreted factor responsible for IL-1β cleavage, we performed size-exclusion chromatography (SEC) on concentrated *S. aureus* SN (Figs. 2*C* and S1*A*). We analyzed the collected fractions for their ability to cleave pro-IL-1β *via* immunoblot, finding cleavage activity across multiple fractions, with fractions six to ten specifically producing the previously described IL-1β species (Fig. 2*D*). IL-1β maturation was also confirmed in these fractions using ELISA, as exemplified for fraction 9 (Fig. 2*E*, see Fig. S1*B for the whole panel*). When examining the biological activity of this fraction toward pro-IL-1β maturation, we again observed strong induction of IL-6 secretion from MeWo cells (Fig. 2*F*, see Fig. S1*C for the whole panel*). Interestingly, fractions 5 and 11 to 15 also produced differentially cleaved IL-1β products, suggesting the presence of additional proteases in the *S. aureus* SN that cleave pro-IL-1β at distinct sites (Fig. 2*D*).

An observation we made during the course of our studies was that keratinocytes cultured in medium used for growing *S. aureus* showed a lower IL-1β signal after stimulation with VbP at various concentrations (Fig. S2*A*). Among the components missing in the *S. aureus* medium was epidermal growth factor (EGF). We wondered if complementing the medium with EGF would enhance inflammasome activation or secretion of IL-1β. Indeed, complementing *S. aureus* medium with recombinant EGF alone restored secretion of IL-1β after inflammasome activation to a similar level as observed with regular medium used for the keratinocytes (Fig. S2*A*). We investigated which inflammasome components were influenced by EGF and blotted for NLRP1 as well as pro-IL-1β. We found that pro-IL-1β as well as cleaved IL-1β levels were diminished in medium lacking EGF but normalized to the same level after complementing the medium with recombinant EGF (Fig. S2, *B* and *C*). This highlights a crucial role of EGF in modulating inflammasome activation and IL-1β secretion in keratinocytes.

### Identification of the cysteine protease and cleavage site of pro-IL-1β

**Figure 3:**
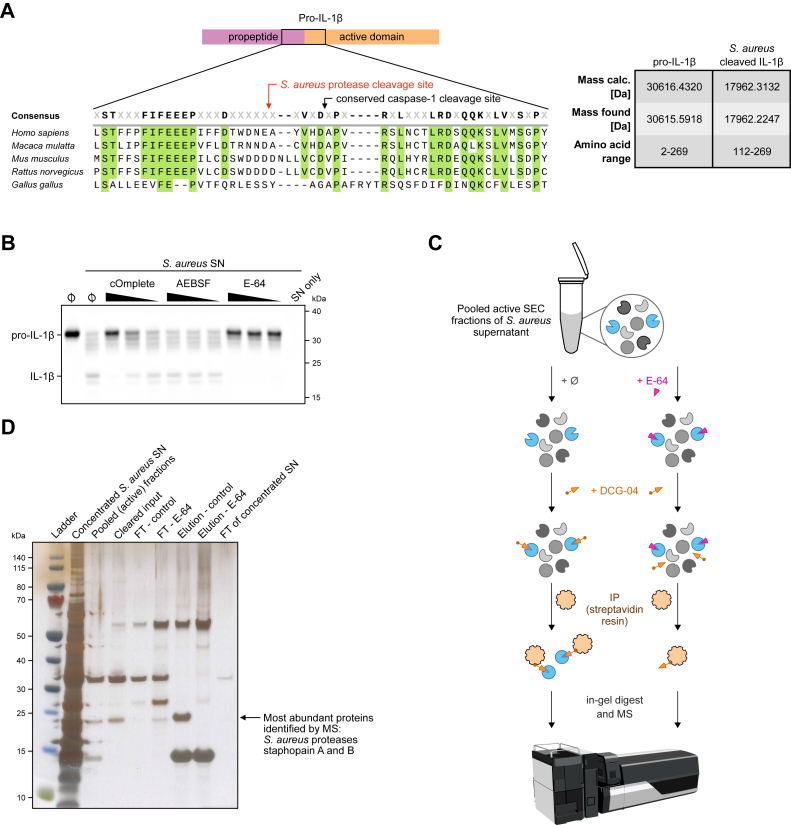
**Pro-IL-1β is cleaved by a cysteine protease at a site proximal to the canonical cleavage site of caspase-1.***A*, recombinant human pro-IL-1β incubated with SEC fraction 9 from Figure 2C was subjected to HPLC and ESI–TOF and cleavage site determined by total mass. Aligned pro-IL-1β sequences of indicated species at the junction between propeptide and active domain are displayed, and conserved amino acid residues are marked. The caspase-1 cleavage site as well as the detected cleavage site of the *Staphylococcus aureus* protease are highlighted. The assigned peptides based on ESI–TOF analysis are depicted next to calculated and found masses. Found masses are shown as experimentally identified uncharged and average masses. *B*, recombinant human pro-IL-1β was left untreated, incubated with *S. aureus* SN, or *S. aureus* SN in combination with indicated protease inhibitors at decreasing concentrations (cOmplete: 1x, 0.1x, and 0.01x; AEBSF and E-64: 50 μM, 5 μM, and 0.5 μM) for 8 h at 37°C. *S. aureus* SN alone (SN only) was used as a control. Assessed inhibitors were cOmplete (serine, cysteine, and metalloprotease inhibitor), AEBSF (serine protease inhibitor), or E-64 (cysteine protease inhibitor). Samples were blotted for IL-1β. *C*, schematic overview of the activity-based profiling principle used to identify the respective cysteine protease, which cleaves IL-1β. Pooled active SEC fractions were either left untreated or pretreated with E-64. In the next step, samples were treated with the activity-based probe for cysteine protease activity DCG-04 (biotinylated version of E-64). Identification of immunoprecipitated proteases was performed by in-gel digest and subsequent mass spectrometry (MS). *D*, silver stain of fractions of activity-based profiling approach from (*C*). *Arrow* indicates protein band subjected to in-gel digest and MS. *S. aureus* cysteine proteases staphopain A and B were among the most abundant proteins identified by MS. Data are depicted as one experiment (*A* and *D*) or as one representative experiment out of two (*B*). ESI, electrospray ionization; FT, flow-through; Pro-IL-1β, inactive precursor of IL-1β; SEC, size-exclusion chromatography.

Next, we aimed to identify the specific cleavage site of pro-IL-1β following treatment with *S. aureus* SN. For this, we subjected both untreated and *in vitro*-cleaved pro-IL-1β to mass spectrometry (MS) analysis. This allowed us to precisely map the cleavage site, revealing that pro-IL-1β was cleaved after amino acid 111, generating an active fragment of 158 amino acids (Fig. 3*A*). This site is located near the consensus cleavage site for caspase-1, which cleaves between amino acids 116 and 117. To validate the identified cleavage site, we generated two pro-IL-1β mutants in which point mutations at positions P2, P1, and P1′ were introduced (pro-IL-1β^N110K, E111P, A112N^ and pro-IL-1β^N110K, E111H, A112N^) (Fig. S3*A*). Recombinant pro-IL-1β^N110K, E111P, A112N^ and pro-IL-1β^N110K, E111H, A112N^ were still cleaved by *S. aureus* SN, but the main cleavage product was now larger in size compared with unmodified pro-IL-1β, suggesting that processing occurred at a different site located closer to the N terminus (Fig. S3*B*). To identify the class of secreted *S. aureus* protease responsible for cleaving pro-IL-1β, we tested various protease inhibitors to assess their ability to block *S. aureus* SN-mediated cleavage. We used cOmplete cocktail, which inhibits serine, cysteine, and metalloproteases, alongside AEBSF, a serine protease inhibitor, and E-64, a cysteine protease inhibitor (Fig. 3*B*). Interestingly, both cOmplete and E-64 inhibited pro-IL-1β cleavage, as detected by immunoblot, indicating that an *S. aureus* cysteine protease, similar to caspase-1, is responsible for this cleavage.

To identify the *S. aureus* protease responsible for cleaving pro-IL-1β, we employed an activity-based probe, DCG-04, a biotinylated version of E-64. Like E-64, DCG-04 covalently binds and irreversibly inhibits cysteine proteases, making it ideal for immunoprecipitation (IP) studies. We incubated pooled active SEC fractions of *S. aureus* SN, leaving some untreated and preincubating others with E-64 to block the active sites of cysteine proteases (Fig. 3*C*). We then performed IP using DCG-04 (Fig. 3*D*). A band corresponding to cysteine proteases was detected only in the untreated sample, which was subsequently analyzed by MS. This approach led to the identification of two cysteine proteases: staphopain A and staphopain B.

### The cysteine protease staphopain A cleaves pro-IL-1β

**Figure 4:**
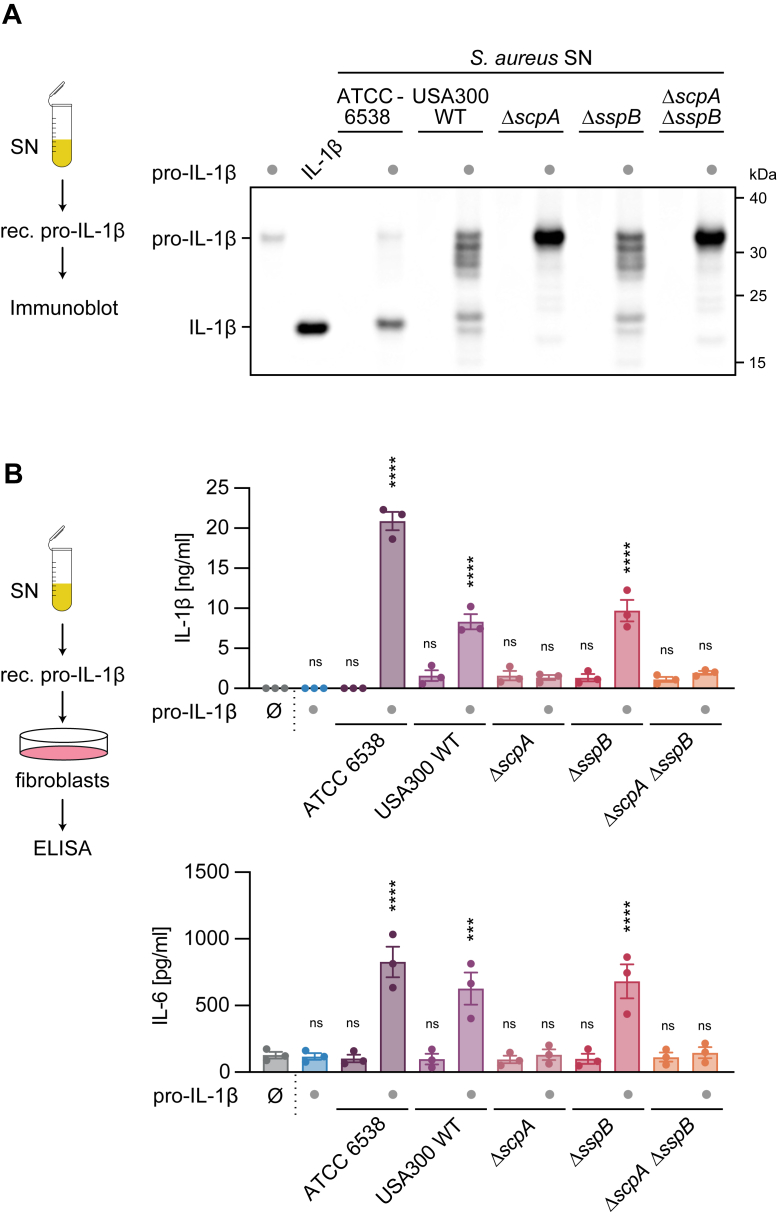
***Staphylococcus aureus* staphopain A, but not staphopain B, cleaves pro-IL-1β.***A*, recombinant human pro-IL-1β (rec. pro-IL-1β) was left untreated or incubated with *S. aureus* SN of indicated *S. aureus* strains for 8 h at 37 °C. In addition, *S. aureus* SN alone of indicated strains was incubated under the same conditions. Recombinant mature IL-1β was used as a control. Samples were blotted for IL-1β. *B*, MeWo fibroblasts were left untreated or stimulated with cleavage products from (*A*). IL-1β and IL-6 levels were determined after 15 h. Data are depicted as one representative experiment out of three (*A*) or as mean ± SEM of *n = 3* independent experiments (*B*). Statistical analysis was conducted by one-way ANOVA with Dunnett’s multiple comparison correction (*B*). Comparisons in (*B*) were made against the untreated control. ∗∗∗∗*p* < 0.0001, ∗∗∗*p* < 0.001; ns, not significant. IL, interleukin; Pro-IL-1β, inactive precursor of IL-1β; SN, supernatant.

Although we identified staphopain A and staphopain B as secreted cysteine proteases from *S. aureus*, it remained unclear which of them, or if both, were capable of cleaving pro-IL-1β. To determine this, we used the USA300 strain of *S. aureus* and its deletion mutants for each protease. In our *in vitro* cleavage assay using recombinant pro-IL-1β, we observed pro-IL-1β cleavage after incubation with SNs from both the *S. aureus* ATCC 6538 strain and the USA300 strain (Fig. 4*A*). Interestingly, while the ATCC 6538 strain yielded one predominant cleavage band, USA300 produced multiple bands, indicating strain-specific expression of proteases. Notably, the predominant cleavage product, resembling IL-1β, was absent in the staphopain A deletion mutant (Δ*scpA*) but remained in the staphopain B deletion mutant (Δ*sspB*). The double deletion mutant (Δ*scpA*/Δ*sspB*) showed no cleavage activity, confirming that staphopain A, and not staphopain B, specifically cleaves pro-IL-1β.

To validate the functional relevance of staphopain A-mediated cleavage, we transferred SNs from *S. aureus* ATCC 6538, USA300 WT, and the corresponding deletion mutants to MeWo cells, following prior incubation with pro-IL-1β. SNs alone served as controls. SNs from both the ATCC 6538 and USA300 WT strains incubated with pro-IL-1β induced IL-6 secretion, whereas pro-IL-1β treated with SNs from the staphopain A (Δ*scpA*) and double mutant (Δ*scpA*/Δ*sspB*) strains did not induce IL-6 secretion (Fig. 4*B*). The measured IL-6 levels correlated with the presence of mature IL-1β detected in the SNs. These findings demonstrate that staphopain A specifically cleaves pro-IL-1β, converting it into its bioactive form.

### *S. aureus* α-toxin mediates cell death of keratinocytes

**Figure 5:**
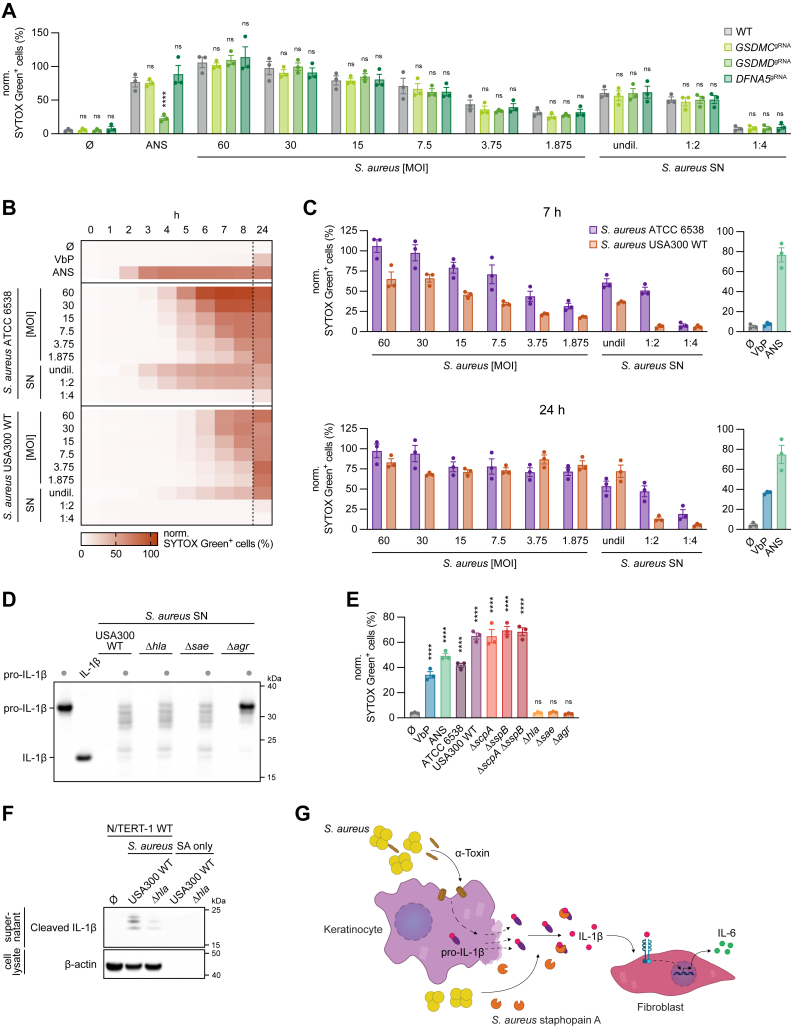
***Staphylococcus aureus* α-toxin mediates cell death of keratinocytes.***A*, keratinocytes with the indicated genotypes were left untreated, stimulated with ANS, infected with *S. aureus* at the indicated MOIs, or stimulated with *S. aureus* SN. SYTOX Green uptake was determined after 7 h. *B*, unmodified keratinocytes were left untreated, stimulated with VbP or ANS, infected with *S. aureus* ATCC 6538 or USA300 WT at the indicated MOIs, or stimulated with *S. aureus* SN from ATCC 6538 or USA300 WT. SYTOX Green uptake was determined at the indicated time points. Note that the control data (Ø and ANS) as well as the *S. aureus* ATCC 6538 data are identical with the WT data shown in (*A*). *C*, time points 7 and 24 h from (*B*) are depicted. *D*, recombinant human pro-IL-1β was left untreated or incubated with *S. aureus* SN of indicated *S. aureus* strains for 8 h at 37 °C. In addition, *S. aureus* SN alone of indicated strains was incubated under the same conditions. Recombinant mature IL-1β was used as a control. Samples were blotted for IL-1β. *E*, unmodified keratinocytes were left untreated, stimulated with VbP or ANS, or stimulated with *S. aureus* SN from indicated *S. aureus* strains. SYTOX Green uptake was determined after 22 h. *F*, unmodified keratinocytes were left untreated or infected with *S. aureus* USA300 WT or α-toxin-deficient mutant (Δ*hla*) for 8 h at MOI 30. Bacteria (SA only) without keratinocytes were used as a control. SNs or lysates were blotted for IL-1β or β-actin. *G*, proposed model for skin infections with *S. aureus*. When *S. aureus* breaches the physical barrier of epidermal cornified keratinocytes, infection of proliferating keratinocytes induces cleavage of pro-IL-1β. Cell death is mediated by the bacterial cell–damaging α-toxin, followed by passive release of pro-IL-1β. Pro-IL-1β is then being cleaved by the secreted *S. aureus* cysteine protease staphopain A. Cleaved and matured IL-1β signals toward its cognate receptor (IL-1R) on dermis-resident fibroblasts, inducing a proinflammatory immune response including IL-6 release. Data are depicted as mean ± SEM of *n = 3* independent experiments (*A*, *C*, and *E*), as mean of *n = 3* independent experiments (*B*), or as one representative experiment out of two (*D* and *F*). Statistical analysis was conducted by one-way ANOVA with Dunnett’s multiple comparison correction (*E*) or two-way ANOVA with Dunnett’s multiple comparison correction (*A*). Comparisons in (*A*) were made against the WT control, whereas comparisons in (*E*) were made against the untreated control. ∗∗∗∗*p* < 0.0001; ns, not significant. ANS, anisomycin; MOI, multiplicity of infection; pro-IL-1β, inactive precursor of IL-1β; SN, supernatant; VbP, Val-boroPro.

While we had established that staphopain A cleaved pro-IL-1β, the mechanism by which the protease gained access to its substrate remained unclear. One potential explanation we considered was that staphopain A might trigger pyroptosis by cleaving GSDMs, such as GSDMD or GSDME, similar to the mechanism by which the group A streptococcal protease SpeB induced pyroptosis (35, 36). RNA-sequencing data from N/TERT-1 keratinocytes showed negligible expression of GSDMA and GSDMB, moderate expression of GSDMC, and high expression of both GSDMD and GSDME (16). These findings raised the possibility that staphopain A could cleave one or more GSDMs, leading to pyroptosis and subsequent release of pro-IL-1β. To test this hypothesis, we generated keratinocyte lines with individual knockouts of GSDMC, GSDMD, or GSDME (gene name: *DFNA5*) and treated them with live *S. aureus* or *S. aureus* SN (Figs. 5*A* and S4, *A* and *B*). However, while ANS induced cell death in a GSDMD-dependent manner, none of the targeted GSDMs were required for *S. aureus*-induced cell death (Fig. 5*A*).

Another possible scenario may involve the *S. aureus* α-toxin, which has been shown to form pores in the host cell membrane (37). This pore formation could lead to cell swelling and subsequent lysis. To investigate this possibility, we utilized the USA300 strain alongside its α-toxin deletion mutant (Δ*hla*). In addition, we included a *sae* regulatory mutant (Δ*sae*), which lacks most secreted toxins and exoenzymes, and an *agr* quorum-sensing mutant (Δ*agr*), in which the production of all toxins and proteases is suppressed. Since all mutants were derived from the USA300 background, we first assessed whether the USA300 and *S. aureus* ATCC 6538 strains induced comparable levels of cell death. After 7 h, infection across various MOIs and stimulation with USA300 SN resulted in less cell death than with the ATCC 6538 strain (Fig. 5, *B* and *C*). However, by 24 h, cell death levels had equalized, indicating that USA300 exhibits delayed cytotoxicity toward keratinocytes compared with ATCC 6538.

To test the role of α-toxin and regulatory pathways, we evaluated the USA300-derived mutants described previously. As expected, SNs from the Δ*hla* and Δ*sae* strains were still capable of cleaving recombinant pro-IL-1β, whereas the Δ*agr* SN showed no cleavage activity (Fig. 5*D*). Furthermore, stimulation of keratinocytes with SNs from the staphopain A and B deletion mutants resulted in robust cell death, comparable to that observed with the WT USA300 strain, indicating that these proteases were not required for cell death induction (Fig. 5*E*). Interestingly, cell death was completely abrogated in cells treated with SNs from the Δ*hla*, Δ*sae*, and Δ*agr* mutants, suggesting a critical role for α-toxin in mediating cytotoxicity (Fig. 5*E*). Consistent with this, infection of keratinocytes with the α-toxin deletion strain resulted in markedly reduced levels of cleaved IL-1β compared with the WT USA300 strain (Fig. 5*F*). These findings indicate that *S. aureus* α-toxin drives cell death, thereby releasing pro-IL-1β, which is subsequently cleaved and activated by staphopain A.

## Discussion

The skin is constantly exposed to environmental bacteria and must maintain a balance between immune homeostasis and immune activation during infections. This balance is crucial for preventing both unchecked bacterial growth and an overactive immune response. Although keratinocytes are not considered classical immune cells, they play a critical role in the skin’s immune defense. Understanding their role in pathogen defense and immune modulation is essential for elucidating the mechanisms of skin infection and inflammation.

In this study, we investigated how keratinocytes respond to the opportunistic pathogen *S. aureus*, with a focus on the key cytokine IL-1β. Normally, IL-1β is activated *via* the inflammasome, which triggers caspase-1 to cleave pro-IL-1β into its mature, active form. Through various complementary approaches, we discovered that *S. aureus* activates IL-1β through an alternative, inflammasome-independent pathway. Our findings revealed that *S. aureus* infection leads to the release of bioactive IL-1β, facilitated by a soluble factor with direct pro-IL-1β cleavage activity. Biochemical analyses identified staphopain A, a secreted protease, as responsible for this cleavage. We found that staphopain A cleaves pro-IL-1β between glutamate (E111) and alanine (A112), located five amino acids upstream of the canonical cleavage site for caspase-1. Mutating positions P2, P1, and P1′ of the identified cleavage site did not abolish the cleavage activity of staphopain A. Instead, pro-IL-1β mutants were cleaved at a different site closer to the N terminus. This observation is consistent with a previous report indicating that staphopain enzymes possess broad cleavage specificity (38). Of note, the identified cleavage site shows high similarity to the known staphopain A cleavage site within the human CXC chemokine receptor 2, where cleavage occurs between aspartate and alanine (39). These findings suggest that inflammasome-independent IL-1β activation by microbial proteases may represent an ancient innate immune mechanism. In fact, *S. aureus* staphopain A is not the only bacterial protease capable of cleaving and activating IL-1β. Other bacterial proteases, such as SpeB from group A streptococci and LasB from *P. aeruginosa*, have similarly been shown to cleave pro-IL-1β near its canonical caspase-1 cleavage site (27, 28). In each case, these proteolytic events generate bioactive IL-1β. This evidence points to a mechanism wherein pro-IL-1β may have evolved as a decoy protein to detect microbial protease activity. It is plausible that pro-IL-1β has adapted to sense bacterial proteolytic activity as part of the broader repertoire of effector-triggered immunity.

Due to significant species divergence between mice and humans in the expression levels of inflammasome proteins in keratinocytes—most notably pro-IL-1β, which is minimally expressed in mouse keratinocytes (40)—it is not feasible to investigate the mechanisms identified in this study using an *in vivo* mouse model. However, indirect evidence from clinical data suggests that IL-1β might play a critical role in controlling *S. aureus* infections, with host-derived IL-1β essential for pathogen containment. Thus, there is evidence to indicate that inhibition of IL-1β in the context of anti-inflammatory regimens may impair the host's immune defense against *S. aureus*. To this end, several studies have reported a notable incidence of *S. aureus* septicemia in patients undergoing anti-IL-1 therapies (41, 42, 43). Furthermore, it is noteworthy that therapy with EGF receptor inhibitors, such as erlotinib, has been associated with compromised host defense mechanisms, particularly against *S. aureus* (44, 45). Consistent with our findings that EGF withdrawal from keratinocyte culture medium results in diminished IL-1β levels, erlotinib-treated keratinocytes exhibit reduced levels of IL-1β expression (45). While these epidemiological data do not conclusively link reduced skin-derived IL-1β responses to these adverse outcomes, the potential connection is plausible and merits further investigation.

Our cytotoxicity studies show that the *S. aureus* pore-forming α-toxin is required to trigger cell death in keratinocytes upon *S. aureus* infection and SN stimulation. Moreover, we found that its role in promoting cell lysis precedes pro-IL-1β cleavage. This observation supports the proposed model that α-toxin-induced pore formation triggers cell swelling and activates the plasma membrane rupture pathway, ultimately leading to cell lysis. This process facilitates the release of intracellular pro-IL-1β into the extracellular space, where it engages with staphopain A, or alternatively allows staphopain A to access the cytosol (Fig. 5*G*).

In summary, our study broadens the understanding of the interplay between keratinocytes and *S. aureus*. Therapeutically, targeting specific proteases responsible for IL-1β cleavage could modulate the inflammatory response and improve outcomes in *S. aureus* infections, opening new approaches for therapeutic intervention. This could be particularly beneficial in conditions where excessive inflammation contributes to disease pathology, such as in atopic dermatitis or chronic wounds.

## Experimental procedures

### Cell culture

N/TERT-1 cells (a gift from J. Rheinwald) were cultured in a 1:2 mixture of Ham’s F12 (catalog no.: 21765029, Gibco) and Dulbecco's modified Eagle's medium (DMEM) (high glucose, no glutamine, no calcium; catalog no.: 21068028, Gibco) supplemented with 1% minimum essential medium (MEM) nonessential amino acid (catalog no.: 11140035, Gibco), 0.5% EpiLife defined growth supplement (homemade), 25 μg/ml bovine pituitary extract (catalog no.: 13028014, Gibco), 30 ng/ml EGF (Max Planck Institute of Biochemistry), 10 mM Hepes (H0887-100ML, Sigma–Aldrich), 2 mM GlutaMAX (catalog no.: 35050038, Gibco), 0.1 mM CaCl_2_ (catalog no.: C7902-500G, Sigma–Aldrich), and 100 U/ml penicillin–streptomycin (catalog no.: 15140122, Gibco). Inflammasome-deficient N/TERT-1 cells have been described previously (14). MeWo cells (a gift from A. Pichlmair) and human embryonic kidney 293T cells were cultivated in DMEM (high glucose; catalog no.: 41965062, Gibco) supplemented with 10% heat-inactivated fetal calf serum (catalog no.: 10270106, Gibco), 1 mM sodium pyruvate (catalog no.: 11360088, Gibco), and 100 U/ml penicillin–streptomycin. All cells were cultured in a humidified incubator at 37 °C with 5% CO_2_.

### CRISPR–Cas9 KO generation with ribonucleoproteins

CRISPR–Cas9 KO generation with ribonucleoproteins was performed as previously described (14). The following sites were targeted: *GSDMC* (5′-CAGAAGCATAAGGCTGACAT-3′/5′-GGAGCATCCATGGTCCACAG-3′), *GSDMD* (5′-CCACGTACACGTTGTCCCCG-3′/5′-ACGCGCACCCACAAGCGGGA-3′), and *DFNA5/GSDME* (5′-TAAGTTACAGCTTCTAAGTC-3′/5′-CAGTTTTTATCCCTCACCCT-3′). Cell lysates of GSDMD- or DFNA5/GSDME-deficient keratinocytes were prepared 7 days after electroporation to check for polyclonal editing by immunoblot. *GSDMC* pool knockout was confirmed using the ICE CRISPR Analysis Tool from Synthego.

### *S. aureus* infections

*S. aureus* subsp. *aureus* Rosenbach bacteria strain ATCC 6538 were grown in *S. aureus* medium (1:2 mixture of Ham’s F12 and DMEM [high glucose, no glutamine, no calcium] supplemented with 1% MEM nonessential amino acid, 10 mM Hepes, 2 mM GlutaMAX, and 0.1 mM CaCl_2_) for 16 h at 37 °C. Bacteria were centrifuged for 10 min at 1200*g* and resuspended in PBS (catalog no.: 14190169, Gibco) for quantification by an asborbance at 600 nm measurement. Bacteria were centrifuged for another 10 min at 1200*g* and resuspended in *S. aureus* medium to reach indicated MOIs. For heat inactivation, bacteria were incubated for 30 min at either 56 °C or 80 °C. Other *S. aureus* strains used in this study were *S. aureus* LAC∗ USA300 CA-MRSA Erm^s^ (AH1263, Boles *et al.* (46)), *S. aureus* USA300 Δ*scpA* (AH1825, Mootz *et al.* (47)), *S. aureus* USA300 Δ*sspB*::pSMUT (AH2594, Mootz *et al.* (48)), *S. aureus* USA300 Δ*scpA* Δ*sspB*::pSMUT (AH2595, Mootz *et al.* (48)), *S. aureus* USA300 *hla*::Tn*551*-Erm (AH1589, Olson *et al.* (49)), *S. aureus* USA300 Δ*saePQRS* (AH2216, Flack *et al.* (50)), and *S. aureus* USA300 *agr*::*tet* (AH1292, Benson *et al.* (51)).

N/TERT-1 cells were plated 24 h prior to infection at 5 × 10^4^ cells/well and 1 × 10^6^ cells/well for 96- and 6-well plates, respectively. N/TERT-1 cells were washed with PBS to remove antibiotics and infected with 80 μl per 96-well or 800 μl per 6 wells at the indicated MOI for 2 h (96-well plate) or 7 h (6-well plate). Subsequently, cells were treated with gentamicin (G1397-10ML, Sigma–Aldrich) by adding 20 μl (96-well plate) or 200 μl (6-well plate) *S. aureus* medium containing gentamicin (final concentration: 50 μg/ml). Cells were incubated for another 16 h (96-well plate) or 1 h (6-well plate), and SNs were harvested. For infections in 6-well plates, cell SNs and lysates were harvested.

### *S. aureus* SN preparation and stimulation

*S. aureus* subsp. *aureus* strain ATCC 6538 and other strains were grown in 10 ml *S. aureus* medium for 16 h at 37 °C. The SN was harvested by centrifugation for 10 min at 1200*g* and sterile-filtered (0.2 μm sterile filter). N/TERT-1 cells were stimulated with *S. aureus* SN (100 μl per 96-well plate or 1 ml per 6-well plate) for 18 h (96-well plate) or 8 h (6-well plate). Samples were harvested as described for *S. aureus* infections.

### Cell stimulation

N/TERT-1 cells were plated at 5 × 10^4^ cells/well and 1 × 10^6^ cells/well for 96- and 6-well plates, respectively. If not otherwise indicated, N/TERT-1 cells in 96-well plates were plated, stimulated with VbP or ANS 28 h later, and SNs were harvested after 14 h. N/TERT-1 cells in 6-well plates were plated in the morning, stimulated with VbP in the evening, and SNs were harvested after 24 h. To investigate EGF depletion from N/TERT-1 medium, N/TERT-1 cells were plated in N/TERT-1 medium in 96- or 6-well plates in the morning, medium was replaced by N/TERT-1 or *S. aureus* medium containing VbP in the absence or the presence of 30 ng/ml EGF in the evening, and SNs (96- and 6-well plates) and cell lysates (6-well plates) were harvested after 24 h. MeWo cells were plated at 5 × 10^4^ cells/well for 96-well plates. MeWo cells were either plated in the morning and stimulated with *in vitro* cleavage products at indicated dilutions in the evening (SNs were harvested after 15 h) or plated in the afternoon and stimulated with *in vitro* cleavage products at the indicated dilutions in the morning (SNs were harvested after 8 h). If not otherwise indicated, stimuli were used at the following concentrations: 2 μM VbP (3719/10, Bio-Techne) and 2 μM ANS (Cay11308-10, Biomol).

### Cell death assay

N/TERT-1 cells were seeded at 5 × 10^4^ cells/well in N/TERT-1 medium (without antibiotics) in 96-well μ-plates (catalog no.: 89626, Ibidi). Twenty-four hours later, cells were infected with *S. aureus* at the indicated MOI or stimulated with *S. aureus* SN at the indicated dilutions as described previously in medium containing 1 μM SYTOX Green Nucleic Acid Stain (S7020; Invitrogen). Cells were imaged at respective time points using a Cellavista Cell Imager (Synentec) equipped with an Olympus 10×/0.30 objective. SYTOX Green uptake was analyzed and quantified using the nuclei count image analysis. Nuclei count data were normalized to the lysis control and shown as normalized SYTOX Green^+^ cells (%).

### ELISA

hIL-1β ELISAs were performed using a homemade ELISA kit (gevokizumab as capture antibody and biotinylated canakinumab as detection antibody) according to the protocol from BD OptEIA Human IL-1β ELISA Set II (catalog no.: 557953, BD Biosciences). hIL-6 ELISAs (catalog no.: 555220, BD Biosciences) and hIL-18 ELISAs (catalog no.: DY318-05, R&D Systems) were performed according to the supplier’s protocol.

### Immunoblotting

Whole-cell lysates were prepared by lysing cells directly in Laemmli buffer and boiling for 5 min at 95 °C. Cell SNs were subjected to methanol–chloroform precipitation (52), and pellets were resuspended in Laemmli buffer and boiled. Samples were separated by denaturing and reducing Bis–Tris SDS-PAGE (Thermo Fisher Scientific), blotted onto 0.2-μm nitrocellulose membranes (catalog no.: 10600004, Cytiva), and blocked in 3% milk (catalog no.: T145.2, Carl Roth). Membranes were incubated with indicated primary and corresponding secondary antibodies. Chemiluminescent signals were recorded with a CCD camera. Antibodies used in this study were anti-NLRP1 (catalog no.: 679802, Research Resource Identifier [RRID]: AB_2566263, BioLegend), anti-ASC (catalog no.: AG-25B-0006-C100, RRID: AB_2490442, Adipogen Life Sciences), anti-Caspase-1 (catalog no.: AG-20B-0048-C100, RRID: AB_2490257, Adipogen Life Sciences), anti-IL-1β (homemade gevokizumab), anti-GSDMD (catalog no.: NBP2-33422, RRID: AB_2687913, Novus Biologicals), anti-DFNA5/GSDME (catalog no.: ab215191, RRID: AB_2737000, Abcam), anti-β-actin-HRP (catalog no.: sc-47778, RRID: AB_2714189, Santa Cruz Biotechnology), goat anti-human IgG-HRP (catalog no.: AP112P, RRID: AB_90720, Sigma–Aldrich), horse anti-mouse IgG-HRP (catalog no.: 7076P2, RRID: AB_330924, Cell Signaling Technology), and goat anti-rabbit IgG-HRP (catalog no.: 7074P2, RRID: AB_2099233, Cell Signaling Technology). All primary antibodies were used at a 1:2000 dilution, whereas all secondary antibodies were at a 1:5000 dilution.

### Fractionation of *S. aureus* SN

*S. aureus* subsp. *aureus* strain ATCC 6538 bacteria were grown in 20 ml *S. aureus* medium for 16 h at 37 °C. The SN was harvested by centrifugation for 10 min at 1200*g*, sterile-filtered (0.2 μm sterile filter), and concentrated using an Amicon Ultra Centrifugal Filter (10 kDa molecular weight cutoff; UFC901096, Merck Millipore). The concentrated SN was subjected to SEC using a Superdex 75 10/300 GL column (flow rate 0.4 ml/min, injection volume 500 μl; catalog no.: 17517401, Cytiva) in the following buffer: 20 mM Hepes (pH 6.9), 50 mM NaCl, and 1 mM DTT. Indicated SEC fractions were used for *in vitro* cleavage of recombinant human pro-IL-1β. Selected SEC fractions were separated by denaturing and reducing Bis–Tris SDS-PAGE and visualized using silver staining.

Protein standards were used as references. Therefore, a gel filtration standard (catalog no.: 1511901, Bio-Rad Laboratories) containing the following components was subjected to SEC using a Superdex 75 10/300 GL column (flow rate 0.5 ml/min, injection volume 500 μl) in PBS: thyroglobulin (670 kDa), bovine γ-globulin (158 kDa), chicken ovalbumin (44 kDa), equine myoglobin (17 kDa), and vitamin B12 (1.35 kDa).

### Silver staining

Samples were mixed with Laemmli buffer, boiled for 5 min at 95 °C, and separated by denaturing and reducing SDS-PAGE. The resulting gels were stained with silver using self-made buffers.

### Purification of pro-IL-1β mutants

Human WT pro-IL-1β and mutants were fused to a C-terminal FLAG-tag, cloned into the pFUGW vector, and transfected into human embryonic kidney 293T cells. For transfection, 36 μg pFUGW plasmid and 98 μg PEI Max (catalog no.: 24765, Polysciences) were incubated separately for 5 min in prewarmed Opti-MEM (catalog no.: 31985047, Gibco). Subsequently, the two reagents were combined and incubated for additional 25 min at room temperature and afterward added to the cells. Cells were harvested after 18 h. All subsequent purification steps were performed at 4 °C. Cells were resuspended in buffer A (20 mM Hepes, pH 7.4, 2 mM MgCl_2_, 150 mM NaCl, 0.1% [v/v] NP-40 + cOmplete Protease Inhibitor [CO-RO; Roche]) and incubated for 1 h. The lysate was cleared by centrifugation at 48000*g* for 15 min. Cleared lysate was incubated with 25 μl Pierce Anti-DYKDDDDK Magnetic Agarose beads (A36797; Thermo Fisher Scientific) pre-equilibrated with buffer A for 2 h. The SN was removed, and beads were washed with 500 μl buffer A three times. For elution, beads were incubated with 100 μl 3X FLAG peptide (0.5 mg/ml, HY-P0319; MedChemExpress) for 1 h. Eluted pro-IL-1β was used for *in vitro* cleavage assays without further purification.

### Coomassie staining of pro-IL-1β

To analyze purity of recombinant human pro-IL-1β mutants, proteins were separated by denaturing and reducing Bis–Tris SDS-PAGE. Gels were stained with Coomassie (45% ethanol, 10% acetic acid, 1 g/l Coomassie Brilliant Blue R-250 Dye in Milli-Q water) for 1 h at room temperature and destained (20% ethanol, 10% acetic acid in Milli-Q water) overnight.

### *In vitro* cleavage assays

Recombinant human pro-IL-1β (2.86 μg/ml; Max Planck Institute of Biochemistry) or mutant pro-IL-1β was digested with SN of an overnight sterile-filtered *S. aureus* culture of indicated *S. aureus* strains or SEC fractions of *S. aureus* ATCC 6538 SN for 8 h at 37 °C in cleavage buffer (20 mM Hepes [pH 6.9], 50 mM NaCl, and 1 mM DTT). Recombinant mature human IL-1β (Max Planck Institute of Biochemistry) was included as a control. To identify the class of secreted *S. aureus* protease, 2.67 μg/ml recombinant human pro-IL-1β was digested with SN of an overnight sterile-filtered *S. aureus* ATCC 6538 culture in the presence of a protease inhibitor. Protease inhibitors were used at the following concentrations: cOmplete Protease Inhibitor Cocktail (1x, 0.1x, and 0.01x; CO-RO; Roche), AEBSF (50 μM, 5 μM, and 0.5 μM; catalog no.: A8456-25MG, Sigma–Aldrich), and E-64 (50 μM, 5 μM, and 0.5 μM; catalog no.: E3132-1MG, Sigma–Aldrich). 6x Laemmli (60 mM Tris [pH 6.8], 9.3% DTT [w/v], 12% SDS [w/v], 47% glycerol [v/v], and 0.06% bromphenol blue [w/v]) were added, and samples were boiled for 5 min at 95 °C and subjected to immunoblotting.

### HPLC–MS analysis of *S. aureus* protease cleavage site in human pro-IL-1β

Recombinant human pro-IL-1β (5 μg) was digested with 2 μg active SEC fraction 9 containing secreted *S. aureus* factors for 8 h at 37 °C in cleavage buffer (20 mM Hepes [pH 6.9], 50 mM NaCl, and 1 mM DTT). As controls, 5 μg recombinant human pro-IL-1β or 2 μg active SEC fraction 9 alone were incubated for 8 h at 37 °C in cleavage buffer.

For MS of intact peptides, an Agilent 1100 HPLC combined with a Bruker Daltonics microTOF mass spectrometer with electrospray ionization–MS was used. Operating parameters were as follows: positive-ion mode, mass range 800 to 3000 *m/z*. For separation, a Phenomenex Aeris 200 Å column pore size (WIDEPORE C4, 3.6 μm, 2.1 × 100 mm) was used. As buffer A, 0.05% TFA in H_2_O (pH 2.0) and as buffer B, 0.05% TFA in acetonitrile (ACN) (pH 2.0), were used. The flow rate was set to 250 μl/min. The gradient was as follows: 20% B (start); 0 → 15 min, transition to 80% B; 15 → 16 min, transition to 95% B; 16 → 18 min, transition to 20% B; 18 min → end, 20% B.

Data were processed using Bruker Daltonics Compass DataAnalysis software, deconvoluted with “MaximumEntropy” and 10000 instrument resolving power.

### IP of cysteine proteases from *S. aureus* SN

*S. aureus* subsp. *aureus* Rosenbach strain ATCC 6538 bacteria were grown in 60 ml N/TERT-1 medium (without antibiotics) for 21 h at 37 °C under steady shaking. Following, the SN was harvested by centrifugation for 10 min at 3000*g* and sterile-filtered (0.2 μm sterile filter). The SN was concentrated using an Amicon Ultra Centrifugal Filter (3 kDa molecular weight cutoff; UFC900324, Merck Millipore), and buffer was exchanged to 20 mM Hepes (pH 6.9), 50 mM NaCl, 1 mM DTT, yielding a final volume of around 500 μl. SEC was performed using a Superdex 75 10/300 GL column (injection volume 500 μl), and active fractions were pooled and concentrated to 3 ml, whereas 1 ml was used for further experiments. The samples were precleared with 40 μl packed Streptavidin resin (L00353, GenScript) and afterward split into two parts. One part was left untreated, and the other part was pretreated with 4 μM E-64 to occupy active sites of cysteine proteases for 2 h at 20 °C. Following, both samples were treated with 20 μM DCG-04 (catalog no.: 530629, MedKoo) and incubated for 7 h at 20 °C. Subsequently, SDS (final concentration: 0.5%) was added to samples to denature proteins and boiled for 5 min at 95°C to make the biotin moiety accessible for IP. Salt exchange was conducted to 20 mM Hepes (pH 6.9), 50 mM NaCl, 1 mM DTT using a HiTrap Desalting column (catalog no.: 29048684, Cytiva). Subsequently, DCG-04-labeled proteins were immunoprecipitated from samples using streptavidin resin for 1 h at 4 °C and washed three times with wash buffer (20 mM Hepes [pH 6.9], 750 mM NaCl, 1 mM DTT, and 0.1% Tween). Samples were either subjected to SDS-PAGE and subsequent silver staining or subjected to MS analysis (in-gel digest).

### In-gel digest of immunoprecipitated cysteine proteases and MS analysis

Cut gel pieces were washed twice with destaining buffer (25 mM ammonium bicarbonate, 50% ethanol) for 20 min at 25 °C and dehydrated with 500 μl ACN at 25 °C until they turned white. Liquid was discarded, and gel pieces were dried in the speed-vac. After rehydration with reduction buffer (10 mM DTT in 50 mM ammonium bicarbonate), gel pieces were incubated for 60 min at 56 °C. Alkylation buffer (55 mM iodoacetamide in 50 mM ammonium bicarbonate) was added, and gel pieces were incubated for 45 min at 25 °C in the dark. Gel pieces were washed in digestion buffer (50 mM ammonium bicarbonate) for 20 min at 25 °C and dehydrated with ACN for 10 min at 25 °C, and these steps were repeated once more. Gel pieces were dried for 20 min in the speed-vac at 45 °C and rehydrated in trypsin solution (12.5 ng/μl trypsin in 50 mM ammonium bicarbonate) for 20 min at 4 °C. Excess trypsin was removed, and digestion buffer was added before overnight incubation at 37 °C. To stop acidification, 2 μl of 100% TFA was added. Peptides were extracted from gel pieces by adding extraction buffer (3% TFA, 30% ACN) and shaking vigorously for 10 min at 25 °C twice. Gel pieces were dehydrated with 100% ACN for 10 min shaking vigorously. Extracted peptides were combined and dried in a speed-vac to remove ACN. Peptides were cleaned up using C18 Stage tips, which were first equilibrated and washed with buffer B (80% ACN, 0.1% TFA) and then buffer A (0.1% TFA) before loading peptides in buffer A. Subsequently, a wash with buffer A and elution in buffer B were performed. Peptides were dried in the speed-vac and resuspended in 2% ACN and 0.3% TFA and injected.

Samples were loaded onto 50 cm columns packed in-house with C18 1.9 μM ReproSil particles, with a Thermo Fisher Scientific EASY-nLC 1000 system coupled to a Thermo Fisher Scientific Q Exactive HFX mass spectrometer. A homemade column oven maintained the column temperature at 60°C. Peptides were eluted with a 120 min gradient starting at 5% buffer B (80% ACN, 0.1% formic acid) followed by a stepwise increase to 30% in 95 min, 60% in 100 min, 95% in 105 min, and 95% in 1 × 5 min at a flow rate of 300 nl/min. Samples were measured in data-dependent acquisition with a TopN MS method in which one full scan (300–1650 *m/z*, *R* = 60,000 at 200 *m/z*, maximum injection time 20 ms) at a target of 3 × 10^6^ ions was first performed, followed by 15 data-dependent MS/MS scans with higher-energy collisional dissociation (automatic gain control target 10^5^ ions, maximum injection time at 20 ms, isolation window 1.4 *m/z*, normalized collision energy 27%, *R* = 15,000 at 200 *m/z*). Dynamic exclusion of 40 s and the Apex trigger from 4 to 7 s was enabled.

The MS raw files were processed by the MaxQuant, version 1.5.0.38 (53) and fragment lists were searched against the *S. aureus* UniProt FASTA databases (2019) with cysteine carbamidomethylation as a fixed modification and N-terminal acetylation and methionine oxidations as variable modifications. We set the false discovery rate to less than 1% at the peptide and protein levels and specified a minimum length of seven amino acids for peptides. Enzyme specificity was set C-terminal to arginine and lysine as expected using trypsin and lysC as proteases and a maximum of two missed cleavages.

### Protein sequence alignment

Protein sequences of pro-IL-1β of *Homo sapiens*, *Macaca mulatta*, *Mus musculus*, *Rattus norvegicus*, and *Gallus gallus* were aligned using the protein sequence alignment tool from SnapGene (version 7.2.1; Dotmatics) running the alignment algorithm MUSCLE (Multiple Sequence Comparison by Log-Expectation), version 3.8.1551.

### Statistical analysis

Statistical significance was determined by either one-way or two-way ANOVA with Dunnett’s correction for multiple testing or unpaired Student’s *t* test. The exact number of replicates (*n*) is shown within figure legends. All statistical analyses were performed using GraphPad Prism 10 (GraphPad Software, Inc).

## Data availability

Raw MS data underlying Figure 3 are deposited in PRIDE database under accession number PXD057289. All data in this study are available within the article, supporting information, and/or from the corresponding author upon request.

## Supporting information

This article contains supporting information.

## Conflict of interest

The authors declare that they have no conflicts of interest with the contents of this article.
