# Supplementary material for: Inflammasome-independent IL-1β activation via staphopain A protease of Staphylococcus aureus[image]
Source: J Biol Chem. 2025 Aug 8;301(10):110574. doi: 10.1016/j.jbc.2025.110574 (PMC12528902; doi:10.1016/j.jbc.2025.110574)
Supplement: Supporting information [file mmc1.pdf]

Supporting information

Inflammasome-independent IL-1β activation via staphopain A protease of *Staphylococcus aureus*

Stefan Bauernfried[1,‡], Tobias Komar[1,‡], Katja Sterle[1], Maria C. Tanzer[2,3,4], Alexander R. Horswill[5], Matthias Mann[2,6], and Veit Hornung[1,*]

[1] Gene Center and Department of Biochemistry, Ludwig-Maximilians-Universität, Munich, Germany
[2] Department of Proteomics and Signal Transduction, Max Planck Institute of Biochemistry, Martinsried, Germany
[3] Walter and Eliza Hall Institute of Medical Research, 1G Royal Parade, Parkville, Victoria 3052, Australia
[4] Department of Medical Biology, University of Melbourne, Parkville, Victoria 3052, Australia
[5] Department of Immunology and Microbiology, University of Colorado Anschutz Medical Campus, Aurora, Colorado, USA.
[6] Novo Nordisk Foundation Center for Protein Research, Faculty of Health and Medical Sciences, University of Copenhagen, Copenhagen, Denmark
[‡] These authors contributed equally to this work.
* For correspondence: Veit Hornung, hornung@genzentrum.lmu.de.

Figures S1-S4

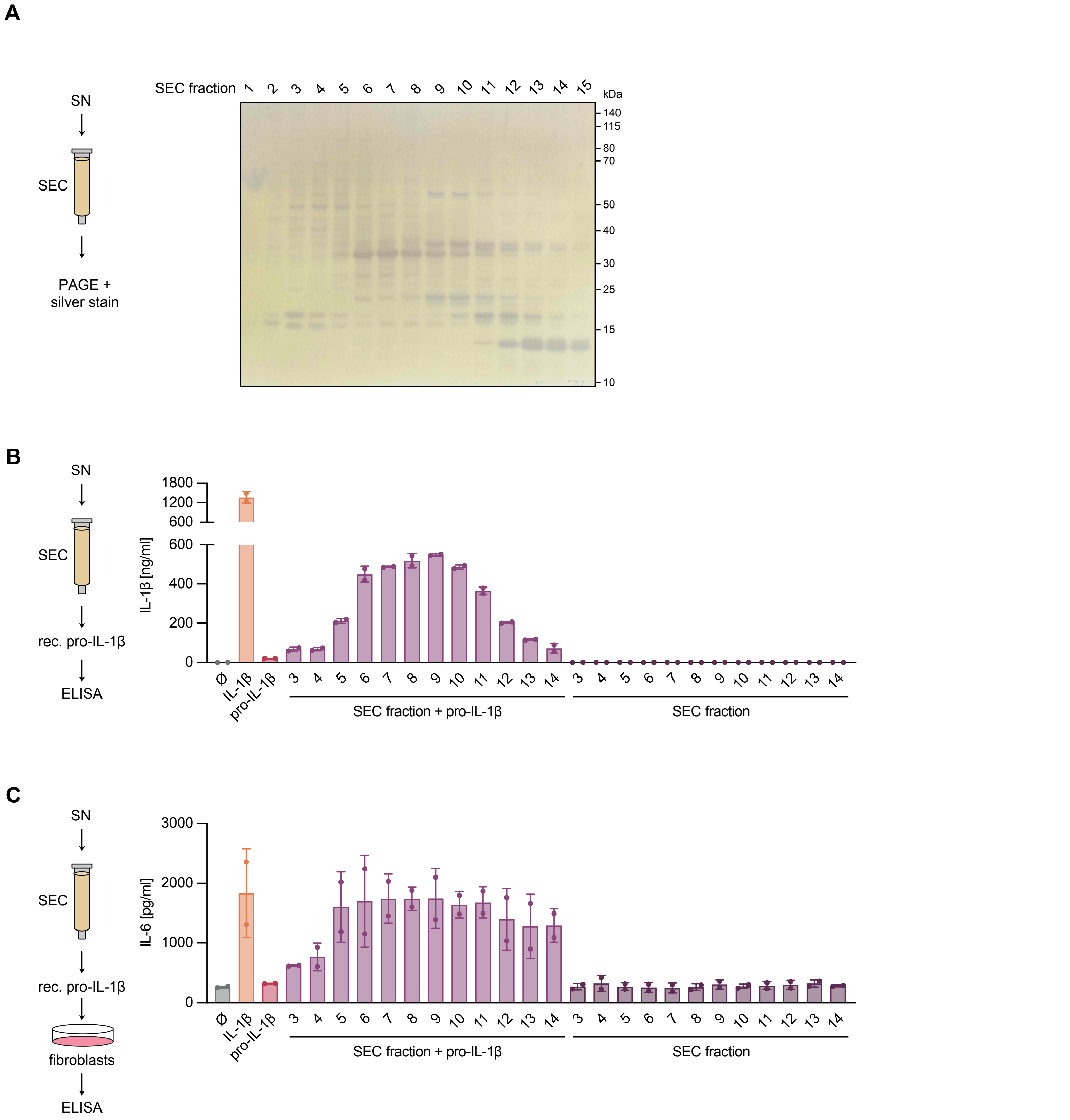

Figure S1: Active SEC fractions of S. aureus supernatant process pro-IL-1β into bioactive proinflammatory cytokine. (A) Concentrated *S. aureus* SN was subjected to size exclusion chromatography (SEC) using a Superdex 75 10/300 GL column. Silver stain of indicated SEC fractions is shown. **(B)** *S. aureus* SN was fractionated by SEC and incubated with recombinant human pro-IL-1β (rec. pro-IL-1β) for 8 h at 37°C. IL-1β levels of rec. mature IL-1β, rec. pro-IL-1β, rec. pro-IL-1β incubated with indicated SEC fractions, or indicated SEC fractions alone were determined. **(C)** MeWo fibroblasts were left untreated or stimulated with cleavage products from (B). IL-6 levels were determined after 8 h. Data are depicted as one representative experiment out of two (A) or as mean ± SD of n = 2 independent experiments (B and C).

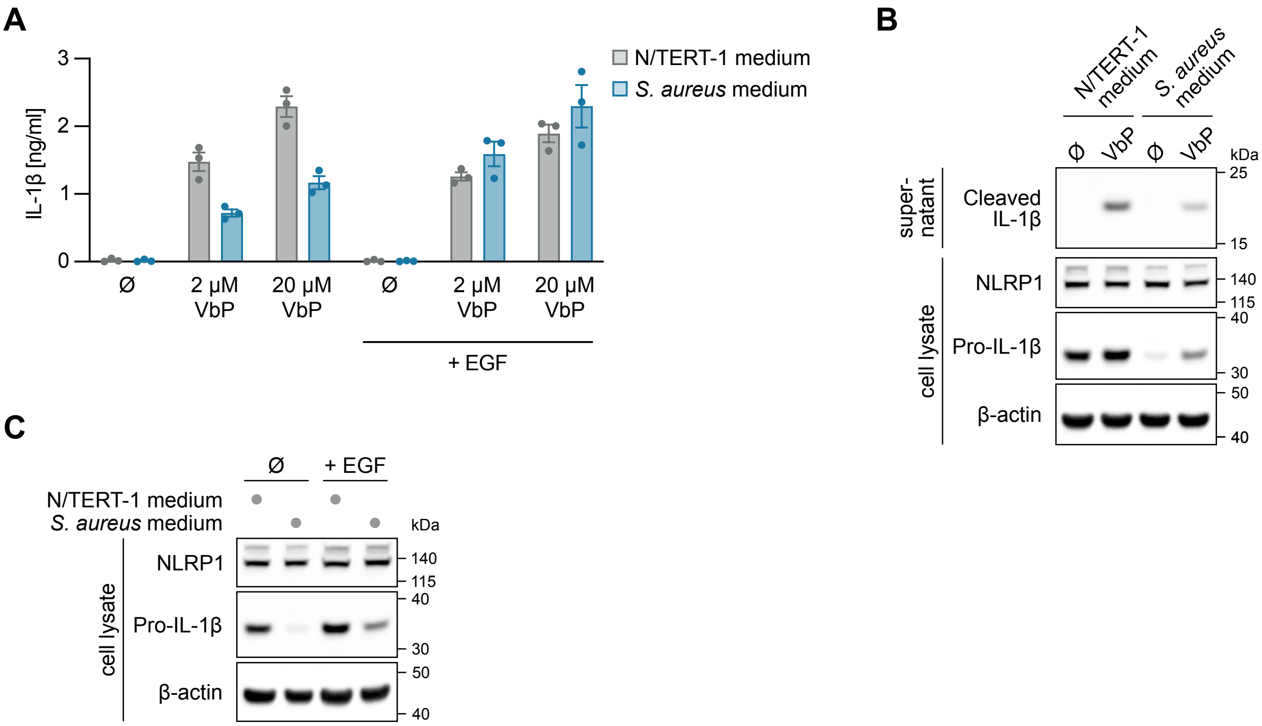

Figure S2: Depleting epidermal growth factor (EGF) from medium reduces pro-IL-1β levels in keratinocytes and thus pro-IL-1β maturation and release. (A) Unmodified keratinocytes cultured in N/TERT-1 or *S. aureus* medium in the absence or presence of additional EGF (30 ng/ml) were left untreated or stimulated with VbP at indicated concentrations. IL-1β levels were determined after 24 h. **(B)** Unmodified keratinocytes cultured in N/TERT-1 or *S. aureus* medium were left untreated or stimulated with VbP. Lysates or supernatants were blotted for NLRP1, IL-1β, or β-actin. **(C)** Unmodified keratinocytes were cultured in N/TERT-1 or *S. aureus* medium in the absence or presence of additional EGF (30 ng/ml) for 24 h. Lysates were blotted for NLRP1, IL-1β, or β-actin. Data are depicted as mean ± SEM of n = 3 independent experiments (A) or as one representative experiment out of two [(B) and (C)].

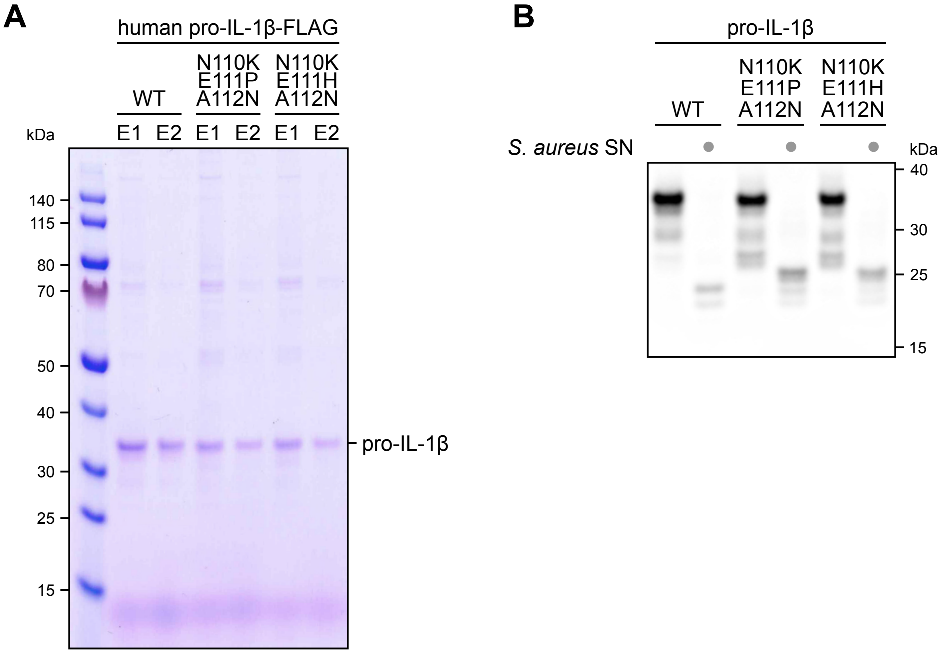

Figure S3: Purification and in vitro cleavage of recombinant human pro-IL-1β mutants by *S. aureus* SN. (A) Coomassie of purified recombinant human WT pro-IL-1β and mutants pro-IL-1β(N110K, E111P, A112N) and pro-IL-1β(N110K, E111H, A112N). Elution fractions 1 (E1) and 2 (E2) are depicted. **(B)** Recombinant human WT pro-IL-1β, pro-IL-1β(N110K, E111P, A112N), or pro-IL-1β(N110K, E111H, A112N) were left untreated or incubated with *S. aureus* SN for 8 h at 37°C. Samples were blotted for IL-1β. Data are depicted as one representative experiment out of two (B).

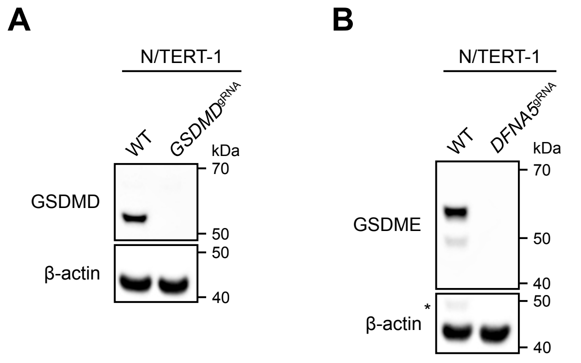

Figure S4: Validation of GSDMD- and DFNA5/GSDME-deficient keratinocytes. (A) Lysates of unmodified or GSDMD-deficient keratinocytes were blotted for GSDMD or β-actin. **(B)** Lysates of unmodified or DFNA5/GSDME-deficient keratinocytes were blotted for GSDME or β-actin. Asterisk denotes band from previous blotting for GSDME. Data are depicted as one representative experiment out of two [(A) and (B)].
